# Three Successful Pregnancies in a Patient with Recurrent Cushing's Disease

**DOI:** 10.1155/2021/5517303

**Published:** 2021-02-13

**Authors:** W. Benothman, G. Saad, M. Kacem, K. Ach

**Affiliations:** Endocrinology Unit, Department of Endocrinology, Farhat Hached University Hospital, Avenue Ibn El Jazzar, Sousse 4000, Tunisia

## Abstract

The association of pregnancy and Cushing's disease (CD) is rare. A 28-year-old woman was admitted for clinical suspicion of Cushing's syndrome. The investigations confirmed the diagnosis of CD with secondary hypogonadotropic hypogonadism due to an invasive pituitary macroadenoma. The patient underwent transsphenoidal adenomectomy, and histopathology showed an adrenocorticotropic hormone pituitary adenoma. Initial remission of CD ensued, and fertility was restored as the patient had 2 uncomplicated pregnancies. Five years and 10 months after surgery, a third spontaneous pregnancy was confirmed with underlying recurrent CD. Having mild hypercortisolism, CD was managed expectantly. The outcome was a healthy full-term neonate with no maternal complications during pregnancy or labor. Our case highlights the challenge faced by physicians of choosing the optimal approach to active CD in pregnancy. In cases where maternal and fetal complications are mild, conservative approach may be used and specific treatment can be postponed until after delivery.

## 1. Introduction

Cushing's disease (CD) is a severe endocrinopathy caused by a pituitary corticotroph adenoma that primarily affects females of childbearing age [1]. However, pregnancy rarely occurs during the course of CD due to the influence of hypercortisolism on the reproductive axis [2]. The best approach to women with active CD who become pregnant is still controversial [3]. Cushing's syndrome (CS) in pregnancy is deemed an obstetric high-risk condition associated with significant morbidity. Therefore, it requires careful surveillance and early effective treatment [4].

We report a case of a patient operated for an invasive pituitary corticotroph macroadenoma with initial remission. She had three successful uneventful pregnancies, the last one being in the setting of CD recurrence.

## 2. Case Report

**Figure 1:**
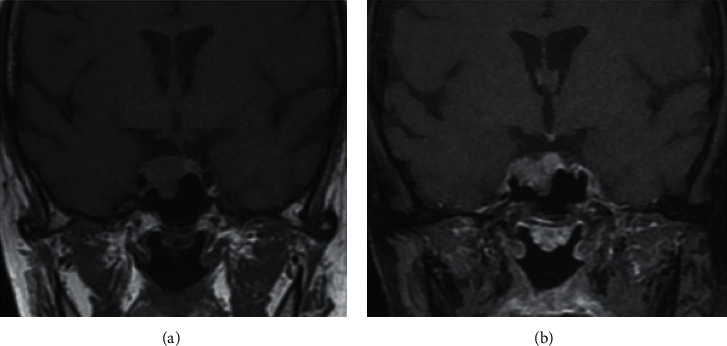
Pregadolinium (a) and postgadolinium (b) T1-weighted coronal images of pituitary MRI showing 17 mm adenoma with right cavernous sinus invasion and sellar floor erosion.

A 28-year-old woman was referred to our endocrinology department by her urologist for clinical suspicion of CS and recently diagnosed diabetes mellitus (DM). She was admitted in the urology department for an acute kidney failure and a urinary tract infection secondary to bilateral ureteral calculi. She had a five-month history of headaches, menstrual irregularity, hirsutism, and depressive symptoms. On physical examination, she had facial plethora, purple abdominal striae, and normal blood pressure. Her body mass index was 22 kg/m^2^. Her laboratory results showed serum potassium concentration at 2 mmol/L, blood glucose level of 17 mmol/L, and both high levels of cholesterol and low-density lipoprotein cholesterol at 6.88 and 4.92 mmol/L, respectively. The patient also had hypercalciuria. Dual X-ray absorptiometry (DXA) confirmed osteoporosis (T-score at −3.2). NPH insulin, psychotropics, and bisphosphonate treatment was started. Her endocrinological workup revealed a plasma adrenocorticotropic hormone (ACTH) level of 152.4 pg/ml (normal <50 pg/ml), morning serum cortisol (MC) of 760 *μ*g/L, 24 h urine-free cortisol (UFC) of 975 *μ*g (normal <45 *μ*g), and unsuppressed cortisol after 1 mg overnight dexamethasone suppression test. Low-dose dexamethasone suppression testing (LDST) showed a plasma cortisol level of 660 *µ*g/L and 24 h UFC of 1500 µg. High-dose dexamethasone suppression testing (HDST) was performed with a postsuppression cortisol level of 280 *μ*g/L (>50% suppression from baseline serum cortisol). The patient showed both normal prolactin level and thyroid function; however, low gonadotropin levels were detected. These results were compatible with a pituitary-dependent CS. Pituitary magnetic resonance imaging (MRI) showed a right 17 mm adenoma with right cavernous sinus invasion and sellar floor erosion. Visual fields were normal (Figure 1).

These investigations confirmed the diagnosis of CD and a secondary hypogonadotropic hypogonadism due to an invasive pituitary macroadenoma. We could not find any sign of an accompanying multiple endocrine neoplasia type 1. Following a three-month pretreatment with ketoconazole (600 mg/day) and cabergoline (2 mg/week), the patient underwent transsphenoidal adenomectomy. Resection was deemed complete by the neurosurgeon. Adenoma cells stained positive for ACTH and were negative for prolactin, luteinizing hormone, follicle-stimulating hormone, growth hormone, and thyroid-stimulating hormone.

Clinical remission of CD ensued, accompanied by a resolution of DM and dyslipidemia and a normalization of both serum potassium and calcium excretion levels. Hormonal tests confirmed the remission with no hypoadrenalism. ACTH, MC, and 24 h UFC levels, at 3 months after surgery, were 15 pg/ml, 110 *µ*g/L, and 60 *µ*g (<4 times the upper limit of normal), respectively. Pituitary MRI showed no persistent mass. The preoperative hypogonadism was functional as gonadotropin levels were normal.

Five and seventeen months after the surgery, she had two spontaneous uneventful pregnancies with successful maternal-fetal outcomes and with no intrapartum or postpartum complications, such as pregnancy-induced hypertension or gestational diabetes. DXA showed improvement of bone density, as T-score was at −1.7. At follow-up, the patient was still at remission, confirmed by a two-year and a half postsurgery hormonal workup. She had regular menstrual cycles. The bone density went back to normal.

Three years and a half after the transsphenoidal surgery, pituitary MRI showed tumor remnant invading the right cavernous sinus. The patient had no clinical signs of CS. However, the endocrine laboratory assessment revealed high ACTH concentration exceeding 100 pg/ml. Plasma cortisol level was not suppressed with LDST (>18 *µ*g/L). In light of these findings, recurrent CD was diagnosed. Shortly after, the patient had an extrauterine pregnancy with an intrauterine device *in situ*. Afterwards, she was lost to follow-up.

**Figure 2:**
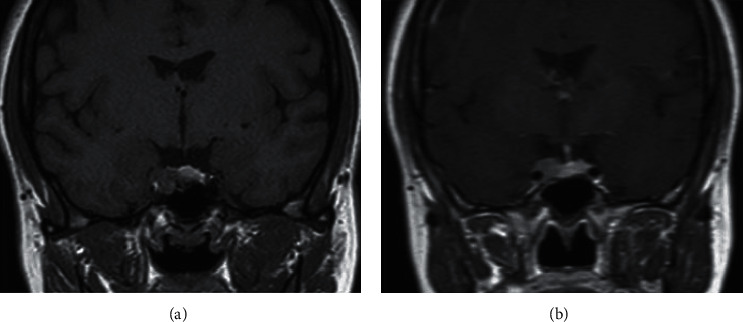
Pregadolinium (a) and postgadolinium (b) T1-weighted coronal images of pituitary MRI showing 10 mm adenoma with right cavernous sinus invasion.

Five years and 8 months after her surgery, she returned for reexamination. She still had no clinical features of hypercortisolemia. Her menstrual cycles were regular. Biochemical workup was as follows: 24 h UFC 150 *µ*g, ACTH 156 pg/ml, and cortisol after 1 mg overnight dexamethasone suppression 20 *µ*g/L. Two months later, on her next visit, the patient reported a confirmed pregnancy at 5 weeks of amenorrhea. The pituitary MRI, performed at 3 weeks of amenorrhea, visualized a macroadenoma remnant measuring 8 × 10 mm invading the right cavernous sinus (grade 4 of Knosp) (Figure 2). All investigations confirmed again the diagnosis of recurrent CD with mild hypercorticism. Due to the risk of maternal complications and the possibility of unpredictable fetal anomalies, therapeutic abortion was discussed as an option, but the patient refused. CD was then managed expectantly.

Throughout her pregnancy, she had no specific treatment, in particular, no dopamine agonists. She had normal blood pressure and manifested no signs of pituitary apoplexy. Laboratory tests were in normal range. Both the oral glucose (75 gr) tolerance test at the 26^th^ week of amenorrhea and the thyroid function were normal. Ultrasound imaging showed normal fetal growth and anatomy with no signs of macrosomia, hydramnios, or intrauterine growth retardation. Monitored closely by her obstetrician and us and at full term, the patient delivered a healthy male infant, weighing 3250 grams, by vaginal delivery.

**Figure 3:**
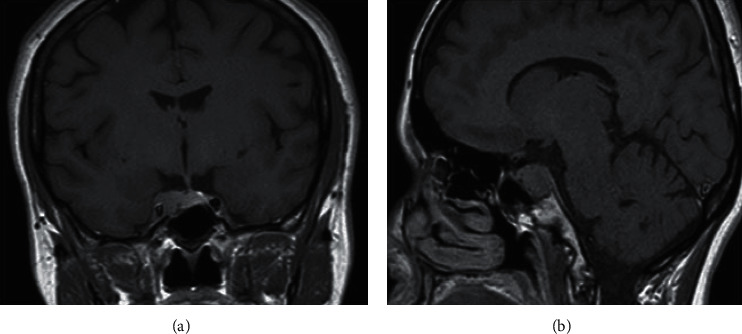
Coronal (a) and sagittal (b) images of pituitary MRI showing 10 mm hypoenhancing adenoma with invasion of the right cavernous sinus.

Three months postpartum, the patient still had clinical “eucorticism,” and she reported that she was breastfeeding. MC was 156 *µ*g/L. Plasma cortisol levels were not suppressed with LDST. The pituitary MRI showed that the adenoma remained stable after pregnancy (Figure 3). A multidisciplinary collaboration between us, neurosurgeons, and radiation oncologists is being arranged for the management of the recurrent CD. Decision will be made after discussion with the patient.

## 3. Discussion

One of the unique aspects of our case is that CD was caused by a pituitary corticotroph macroadenoma. ACTH-secreting macroadenomas account for only about 6% of all CD cases [5]. Initial remission was achieved with a transsphenoidal surgery. Shortly after, fertility was restored and the patient had 2 uncomplicated pregnancies. As the diagnosis of CD recurrence was established, a third pregnancy was confirmed. Adding another unusual aspect to our case, pregnancy is exceptionally rare in CS. The first case was reported in 1953 by Hunt and McConahey [6]. More than 200 cases have been reported in the literature since then [7]. The most frequent etiology of CS diagnosed during pregnancy is adrenal adenoma (40 to 60% of the cases), whilst in nonpregnant women, it is pituitary lesions (CD in 70% of the cases). Such difference is still poorly understood [8, 9]. It has been suggested that hypersecretion of both cortisol and androgens in CD is associated with impaired fertility, in contrast to an almost exclusive hypersecretion of cortisol with minimal androgen production in peripheral CS [10].

It is normal to observe changes in the pituitary gland and endocrine function during pregnancy. Often extending into the suprasellar space towards the optic chiasm, the pituitary gland increases nearly 1.5 times in size [11]. The increased sensitivity of pituitary corticotrophs to CRH, their decreased sensitivity to cortisol's negative feedback inhibition, and a placental CRH production are responsible of the rise of maternal plasma ACTH and cortisol. Plasma concentrations of both hormones typically reach levels comparable to those observed in patients with CD and increase during labor [12, 13]. This transient, but physiologic, relative hypercortisolism during gestation and the immediate postpartum period may somehow promote tumorigenesis resulting in a higher risk of ACTH-secreting pituitary adenomas and CD following pregnancy [13]. In an exploratory study by Palejwala et al. [14], over one-fourth of all women of reproductive age with CD appeared to have symptomatic disease onset within 1 year of childbirth. The median number of pregnancies in the pregnancy-associated CD cohort was 2 suggesting that repeated “exposure” to the stress of pregnancy may also be a significant factor. Two thirds of women of reproductive age and nonpregnancy-associated CD had no pregnancies prior to their diagnosis of CD. This fact, and that CD affects both sexes, strongly advocates that the gestational changes may be just one possible factor among many others that ultimately leads to a clinically significant corticotroph adenoma.

In light of these findings, the factors in our case of a recurrent CD are the repeated exposure to pituitary corticotroph hyperactivity during 2 peripartum periods and essentially the likely incomplete surgical resection. Even though exeresis was deemed complete by the neurosurgeon and early postsurgery imaging, it is greatly difficult to obtain such result for macroadenomas. The subsequent growth of remnant tumor cells was responsible for the relapse of the hypercortisolism. Extensive experience on nonpregnant patients with CD has noted that, following transsphenoidal surgery, a considerable proportion of individuals has normal plasma and urinary levels of cortisol without developing adrenal insufficiency, just as our case. The clinical symptoms improved but, in most cases, hypercortisolism reappeared during follow-up [15]. Actuarial recurrence rates of CD after initial successful transsphenoidal surgery at 1, 2, 3, and 5 years were 0.5, 6.7, 10.8, and 25.5%, respectively [16]. The diagnosis of recurrence was confirmed three years and a half after the surgery for our patient, roughly 2 years after her last pregnancy. It is quite possible that the gestational hypercortisolemia was responsible of an earlier recurrence, as the adenoma remnant was stimulated by the hormonal milieu during the two pregnancies.

Approximately 40% of patients with CD during pregnancy received treatment during gestation. Of these, 11.3% women only received medical treatment [8]. Surgery is the first-line treatment for CD in pregnant and nonpregnant patients, with reported live birth rates up to 87% following transsphenoidal adenomectomy or adrenalectomy [4]. Due to their potential harmful or teratogenic effects and their delayed results, other options to treat hypercortisolism, such as radiotherapy and mitotane, are contraindicated during gestation [17]. In all cases of successfully treated CD with transsphenoidal surgery, the procedure was performed between the end of the first trimester and the early second trimester, an optimum period with lower maternal and fetal complications. Medical therapy is a second treatment option, generally initiated during the second or third trimesters. The most common steroidogenesis inhibitors were metyrapone followed by ketoconazole and cyproheptadine used in 61%, 15%, and 11% of cases, respectively [4]. Dopamine agonists, the only available molecules in Tunisia, have been successfully used for CD in minority of pregnant patients with no major adverse effects noted [8]. Testing more than 6,000 pregnancies, the safety of bromocriptine in pregnancy, both for the mother and the fetus, was demonstrated. The few studies regarding the safety of cabergoline in pregnancy showed no higher rate of maternal-fetal complications and it is currently an FDA pregnancy category B drug [18]. The stage of pregnancy, etiology, severity of hypercortisolism, and potential benefit of treatment, all contribute to treatment decision-making [2]. Pregnant women with mild hypercortisolism, as our patient, and/or diagnosed late in gestation may be managed expectantly, and causal treatment deferred until after delivery. Potential risks of surgical or medical treatment might exceed the benefits. Pregnancy during CD presents great maternal-fetal risks. Metabolic and cardiovascular parameters should be monitored closely, and hyperglycemia or hypertension should be treated appropriately upon diagnosis [19, 20]. However, some authors suggested that treatment of mild hypercortisolism prevents adverse outcomes, as it increases live birth rate, avoids disease worsening, and reduces the risk of preeclampsia [12]. The most frequently stated maternal complications in CS are hypertension (68%), diabetes (25%), preeclampsia (14%), osteoporosis and fractures (5%), heart failure (3%), and maternal death (2%) [21, 22]. Due to placental enzymes such as 11*β*-hydroxysteroid dehydrogenase type 2, the fetus is normally protected from maternal hypercortisolism. The enzyme converts glucocorticoids to inactive metabolites [23]. However, complications can occur, and the most common ones include prematurity (43%), intrauterine growth restriction (21%), stillbirth (6%), spontaneous abortion or intrauterine death (5%), and hypoadrenalism (2%) [21].

It has been stated that maternal and fetal outcomes are better in patients with CD than in those with peripheral CS [24]. Furthermore, just like in our case, uncomplicated pregnancies ending with vaginal delivery have been reported in women with CS, especially in those treated with no complete remission. It appears that morbidity is rather related to the severity of underlying hypercortisolism [19]. As hypercortisolism was mild in our case, no such complications had been noted.

The pituitary gland rapidly returns to its normal size after the immediate postpartum period, regardless of breastfeeding status [25]. The pituitary MRI showed that the adenoma remained stable after pregnancy for our patient. The choice of treatment method for the recurrent CD will be discussed in a multidisciplinary concertation meeting.

## 4. Conclusion

Pregnancy with preexisting CD poses a challenge to physicians because of its association with maternal and fetal morbidities. The decision on treating CD during pregnancy and choice of treatment method must be approached on a case-by-case basis. A multidisciplinary approach consisting of endocrinology, obstetrics, endocrine surgery, and anesthesiology is highly recommended. A conservative approach may be used and a specific treatment can be postponed until after delivery in cases with mild maternal-fetal complications.

## Data Availability

All data used in this case are available from the corresponding author upon reasonable request.
